# Transcranial magnetic stimulation as a translational biomarker for AMPA receptor modulation

**DOI:** 10.1038/s41398-021-01451-2

**Published:** 2021-05-27

**Authors:** Patricio O’Donnell, Francis M. Dijkstra, Ugur Damar, Lei Quanhong, Annika A. de Goede, Lin Xu, Andres Pascual-Leone, Derek L. Buhl, Rob Zuiker, Titia Q. Ruijs, Jules A. A. C. Heuberger, Paul MacMullin, Martin Lubell, Mahnaz Asgharnejad, Venkatesha Murthy, Alexander Rotenberg, Gabriel E. Jacobs, Laura Rosen

**Affiliations:** 1 Takeda Pharmaceuticals International, Inc., Cambridge, MA USA; 2 McLean Hospital, Department of Psychiatry, Harvard Medical School, Belmont, MA USA; 3 Centre for Human Drug Research (CHDR), Leiden, The Netherlands; 4 Department of Psychiatry, Leiden University Medical Center, Leiden, The Netherlands; 5 Neuromodulation Program, Department of Neurology and F.M. Kirby Center for Neurobiology, Boston Children’s Hospital, Harvard Medical School, Boston, MA USA

**Keywords:** Physiology, Biomarkers, Biomarkers

## Abstract

TAK-653 is a novel α-amino-3-hydroxy-5-methyl-4-isoxazolepropionic acid receptor (AMPAR)-positive allosteric modulator being developed as a potential therapeutic for major depressive disorder (MDD). Currently, there are no translational biomarkers that evaluate physiological responses to the activation of glutamatergic brain circuits available. Here, we tested whether noninvasive neurostimulation, specifically single-pulse or paired-pulse motor cortex transcranial magnetic stimulation (spTMS and ppTMS, respectively), coupled with measures of evoked motor response captures the pharmacodynamic effects of TAK-653 in rats and healthy humans. In the rat study, five escalating TAK-653 doses (0.1–50 mg/kg) or vehicle were administered to 31 adult male rats, while measures of cortical excitability were obtained by spTMS coupled with mechanomyography. Twenty additional rats were used to measure brain and plasma TAK-653 concentrations. The human study was conducted in 24 healthy volunteers (23 males, 1 female) to assess the impact on cortical excitability of 0.5 and 6 mg TAK-653 compared with placebo, measured by spTMS and ppTMS coupled with electromyography in a double-blind crossover design. Plasma TAK-653 levels were also measured. TAK-653 increased both the mechanomyographic response to spTMS in rats and the amplitude of motor-evoked potentials in humans at doses yielding similar plasma concentrations. TAK-653 did not affect resting motor threshold or paired-pulse responses in humans. This is the first report of a translational functional biomarker for AMPA receptor potentiation and indicates that TMS may be a useful translational platform to assess the pharmacodynamic profile of glutamate receptor modulators.

## Introduction

According to the Global Burden of Diseases, Injuries, and Risk Factors study in 2017^1^ depressive disorders such as major depressive disorder (MDD) are amongst the leading causes for years lived with disability (YLD) worldwide. In 2017, depressive disorders were estimated to affect over 264 million people worldwide^1^. Furthermore, depressive disorders are associated with an increased risk of mortality^2^. Pharmacological treatments targeting monoaminergic neurotransmission are available, but these fail to achieve an adequate response in up to 50% of MDD patients^3^. This illustrates the need for the development of novel pharmacological therapeutics for the treatment of MDD.

In 2000 it was demonstrated that subanesthetic doses of ketamine, an *N*-methyl-D-aspartate-receptor (NMDAR) antagonist, had antidepressant effects in patients with depression^4^. Since then, many studies have replicated these findings^5,6^. Although the mechanisms by which ketamine exerts its antidepressant effects are not yet fully understood, it has been demonstrated that NMDAR blockade leads to a selective reduction in γ-aminobutyric acid (GABA) interneuron function that enhances glutamate function and increases α-amino-3-hydroxy-5-methyl-4-isoxazolepropionic acid receptor (AMPAR)-mediated signaling^7^. This leads to a release of brain-derived neurotrophic factor (BDNF) and stimulation of mammalian target of rapamycin (mTOR) signaling, which are both hypothesized to play a role in the pathophysiology of depressive disorders^7^. The importance of AMPA receptors for the antidepressant effects of ketamine was further demonstrated by the finding that pretreatment with an AMPA antagonist completely blocked the antidepressant effects of ketamine^8^. It is therefore hypothesized that direct AMPA receptor potentiation might lead to similar antidepressant efficacy as ketamine, without causing the psychotomimetic side effects commonly observed after ketamine administration^9^.

TAK-653, also known as NBI-1065845, is a central nervous system (CNS)-penetrant, selective AMPA receptor-positive allosteric modulator that is being developed as a potential adjunctive therapeutic agent for patients with MDD^10^. It is intended to enhance or reproduce ketamine-driven AMPAR potentiation. In cognitive and depression-related behavioral assays, TAK-653 exhibited antidepressant-like effects at low exposures in rodents (Haruride Kimura, Takeda Pharmaceuticals, 2019, unpublished data), but evidence of immediate pharmacodynamic (PD) effects that could be translated to human studies was missing. A first-in-human dose-escalating study in healthy volunteers established the safety and tolerability of TAK-653, but there was no established methodology to assess CNS-target engagement or PD effects^11^. To continue TAK-653 development with confidence, we needed a neurocircuit-based translational PD biomarker that captures the modulation of glutamatergic synapses.

The paucity of reliable translational biomarkers that capture functional modulation of brain circuitry is a key challenge in the field of neuropsychiatric drug development^12^. With the exception of evoked potentials sensitive to *N*-methyl-D-aspartate (NMDA) receptor function, such as mismatch negativity and auditory steady-state responses^13–15^, there has been little progress toward translatable glutamate-sensitive circuitry function biomarkers. Reliable PD biomarkers are needed to evaluate the functional impact of novel glutamatergic drugs on defined neurocircuits in order to guide dose selection in clinical studies and to support go/no-go decisions during drug development^12,16^.

Here, we explored whether transcranial magnetic stimulation (TMS) could be applied to produce a translational neurocircuitry biomarker for the development of a novel glutamatergic compound. TMS is a noninvasive neurostimulation method based on the principles of electromagnetic induction, in which a fluctuating magnetic field generates a localized intracranial electric current that can be sufficient to depolarize cortical neurons and activate neuronal circuits^17^. When delivered over the motor cortex, TMS leads to reliable limb muscle activation that can be quantified by surface electromyography (EMG) in humans or by accelerometer-based mechanomyography (MMG) in rats. Motor cortex TMS thus enables measures of input–output relationships between the strength of the cortical electrical stimulus and the magnitude of muscle activation. Using various stimulation paradigms that include single or paired pulses (spTMS and ppTMS, respectively), cortical signals involving glutamate or GABA signaling can be isolated^18–20^. The motor responses to TMS have been well characterized and used to demonstrate that such evoked responses are sensitive to pharmacological manipulation of CNS targets^21,22^. We therefore utilized TMS to assess corticospinal and intracortical excitability, allowing determination of a functional outcome of AMPA receptor activation.

The overall aim of our study was to evaluate TMS-evoked motor responses as potential translational neurocircuitry biomarkers for AMPA receptor modulation by TAK-653. To obtain measures of cortical excitability, we coupled TMS with MMG in rats and EMG in humans. We hypothesized that TMS-evoked motor responses would be amplified by positive allosteric modulation of AMPA receptors by TAK-653. As there was no precedent of TMS use to test the effects of agents that increase glutamate function, we included an open-label ketamine period intended to establish assay sensitivity, based on a report of ketamine effects on TMS in a small sample of healthy volunteers^23^; however, a subsequent report did not show the same effect^24^. Given these mixed results of ketamine on TMS, we did not intend to compare the ketamine results with placebo or TAK-653, so ketamine pharmacokinetic (PK) and TMS results are not be included in this report. The primary goal of the study was to assess neurostimulation with TMS as a translational biomarker for the modulation of excitatory neural circuits.

## Materials, subjects, and methods

### Animals

Adult male Sprague Dawley rats were housed in standard cages in a temperature-controlled facility with a 12 h light/dark cycle and a continuous supply of water and food ad libitum. All procedures were approved by, and in accordance with the guidelines of, the Institutional Animal Care and Use Committee at Boston Children’s Hospital and the National Institutes of Health Guide for the Care and Use of Laboratory Animals. All efforts were made to minimize the number of rats used in the present experiments.

### Dosing and pharmacokinetic assessment in rats

TAK-653 was provided by Takeda Pharmaceutical Company Limited (Japan) and prepared in a vehicle formulation consisting of 0.5% methylcellulose in double-distilled water. All dosing was performed per os via oral gavage in 10 ml/kg. Animals in the vehicle group received an equal volume per weight of the vehicle solution.

We used 20 rats to assess plasma and brain levels of TAK-653 (*n* = 5 per TAK-653 dose). Two hours after TAK-653 administration (0.3, 1, 8, and 50 mg/kg, oral gavage), we collected plasma (intracardiac-blood sampling) and brain (decapitation) specimens.

### TMS in rats

**Fig. 1:**
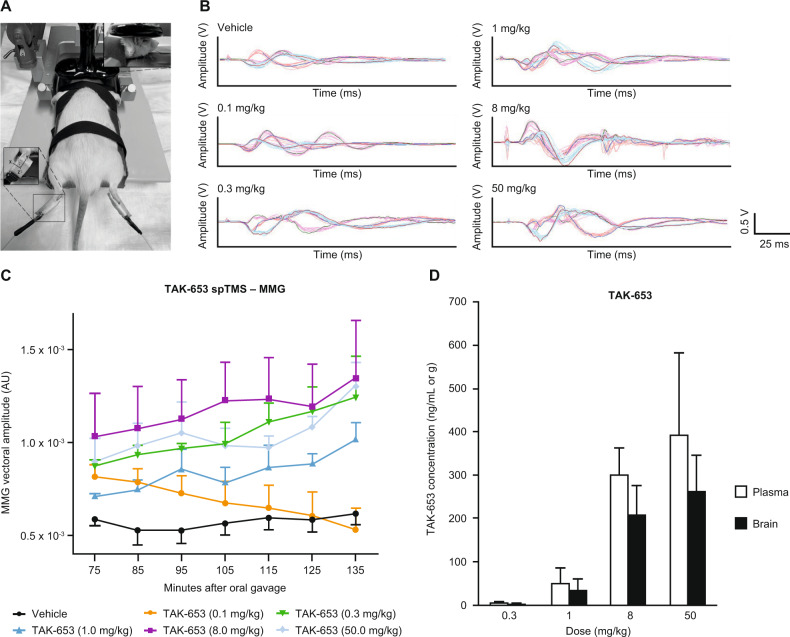
TAK-653 enhanced TMS-evoked motor responses in rats. **A** Example of an animal placement on the stereotaxic apparatus, a small figure-eight coil, and an accelerometer attached to the hind paws. **B** Representative waveforms in three dimensions (pictured in different colors) used for MMG calculation with vehicle or five different doses of TAK-653. **C** Summary graph illustrating the MMG vectoral amplitude over time for all six treatment groups (mean ± SEM). All TAK-653 doses except for 0.1 mg/kg increased MMG amplitude. No dose–response effect was observed. **D** Plasma and brain TAK-653 levels 2 h after administration via oral gavage in rats with the four effective doses. MMG, mechanomyography; spTMS, single-pulse transcranial magnetic stimulation; TMS, transcranial magnetic stimulation.

We tested whether TAK-653 augments corticospinal excitability using spTMS in 31 rats. MMG was chosen instead of needle EMG to allow for a lighter anesthesia level and avoid pain that could confound motor-evoked potential (MEP) responses. Changes in MMG amplitude were captured using three-axis accelerometers attached to the rats’ hind paws^25^. Rats received vehicle (*n* = 6) or TAK-653 (0.1, 0.3, 1, 8, or 50 mg/kg; *n* = 6, 4, 5, 5, and 5, respectively) before being anesthetized with pentobarbital (25 + 15 mg/kg intraperitoneally, doses spaced 30 min apart to maintain stable anesthesia). Because of the oral dosing required for TAK-653, no baseline TMS values were obtained. After appropriate depth of anesthesia was confirmed, rats were placed on a platform and restrained using Velcro straps, and three-axis accelerometers were attached to the soles of the hindlimbs to record MMG (Fig. 1A). spTMS was delivered with a figure-eight coil (25 mm diameter; Magstim, Eden Prairie, MN, USA) centered over the midsagittal plane at the interaural line at which similar bilateral hindlimb activation can be reliably produced. TMS-MMG took place 75–135 min after TAK-653 administration (seven time points, 10 min intervals). Ten single pulses at 80% of the maximum machine-output intensity were applied at each time point.

### Statistical analysis in the rat study

The MMG signals were converted to voltage values, and three-dimensional vector amplitudes were calculated post hoc [√(x^2^ + y^2^ + z^2^)]. Data from animals with 6/10 or more MMG signals with acceptable quality (obtained during rest, in absence of baseline muscle activity) at all seven time points in at least one hindlimb were used for analysis. Data were analyzed in GraphPad Prism (Version 8.0.3, GraphPad Software, San Diego, CA). A mixed-model repeated-measures 2-way analysis of variance (ANOVA) was used to compare the effects of different TAK-653 doses and vehicle on TMS measures. Time and dose were fixed factors and animal was a random factor.

### Human study participants and design

This study was approved by the Medical Ethics Committee, Foundation Beoordeling Ethiek Biomedisch Onderzoek (BEBO) and was registered at clinicaltrials.gov (NCT03792672). Written informed consent was obtained from all participants before study start. The study was performed according to International Conference on Harmonisation guidelines on Good Clinical Practice guidelines, as laid down in the Declaration of Helsinki and its latest amendments. The study was sponsored by Takeda Pharmaceuticals and conducted at the Centre for Human Drug Research, Leiden, The Netherlands, from January 23, 2019 to June 18, 2019. The study was registered at Clinicaltrials.gov; http://ClinicalTrials.gov; NCT03792672.

**Fig. 2:**
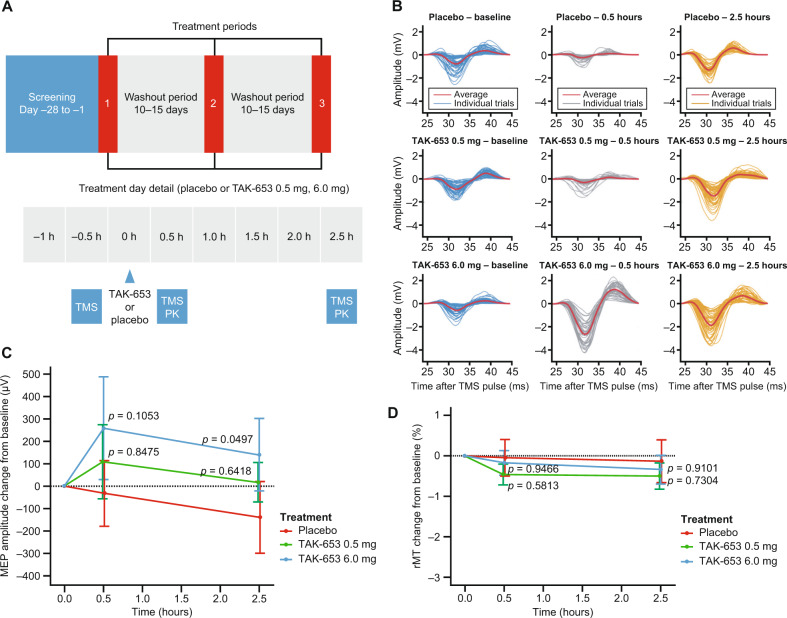
6 mg TAK-653 enhanced MEPs in healthy volunteers. **A** Study schematic (top) and detail of treatment day events (bottom). **B** representative MEP waveforms from one participant at baseline, 30 min, and 2.5 h post-dose for all three treatment periods. **C** Changes from baseline in MEP amplitude for placebo, 0.5 mg TAK-653, and 6 mg TAK-653 periods. *p* = Dunnett adjusted *p* value. **D** Changes from baseline in rMT for all three periods. MEP, motor-evoked potentials; PK, pharmacokinetic; rMT, resting motor threshold; TMS, transcranial magnetic stimulation.

The study consisted of an initial randomized, double-blind, placebo-controlled, three-period crossover phase, followed by an open-label ketamine period. The crossover phase included three treatments (oral placebo, TAK-653 0.5 mg, and TAK-653 6 mg), each 1 day in duration, with washout periods of 10–15 days (Fig. 2A**)**. During treatment days, participants reported at the research center in the morning. Prior to dosing, safety assessments were performed, consisting of physical examination, urine drug screen, urinalysis, vital signs, electrocardiogram, and safety chemistry and hematology laboratory assessments. TMS–EMG assessments were performed 40 min prior to dosing (baseline), 30 min after dosing, and at expected *t*_max_ (2.5 h after dosing). Participants were discharged by a physician 6 h after dosing.

Healthy males and females (of non-childbearing potential) between 18 and 55 years of age were selected. Participants with contraindications for TMS procedures based on the TMS safety questionnaire^26^ (such as having metal objects in the brain or having a family history of epilepsy, seizures, or convulsions) were excluded. Individuals with a resting motor threshold (rMT) higher than 75% of the maximum stimulator output (MSO) were excluded, as stimulation at 120% of this value would be very close to the MSO. Participants having a clinically significant previous or current psychiatric disorder according to the Diagnostic and Statistical Manual of Mental Disorders, Fifth Edition (DSM-5) or a history of alcohol consumption exceeding two standard drinks per day on average were also excluded. Participants were not allowed to use concomitant medications from 7 days before administration of the first dose of study drug throughout the study. Use of alcohol was not allowed from 7 days before the screening visit and 7 days before dosing until the last treatment period. Participants refrained from using caffeine from 24 h before the screening visit, 24 h before each dosing, and during each treatment period. In between visits, participants were allowed up to six servings of caffeine per day. From 48 h before each dosing until the end of the treatment period, participants were not allowed to smoke. In between visits, participants were allowed to smoke up to five cigarettes a day.

### Human pharmacokinetic assessment

Pharmacokinetic assessment times for TAK-653 were matched to the times of TMS procedures. For TAK-653, samples were collected pre-dose, and 30 min and 2.5 h post-dose. Plasma concentrations of TAK-653 were measured by a validated high-performance liquid chromatography with tandem mass spectrometry assay, and the lower limit of quantitation was 0.1 ng/ml.

### Human TMS

TMS measurements were conducted using a MagPro R30 with MagOption stimulator and an MCF-B65 butterfly coil (2 × 75 mm; both MagVenture GmbH, Hueckelhoven, Germany). The motor cortex of the dominant hemisphere, as assessed by the Edinburgh Handedness Questionnaire^27^, was stimulated to elicit a motor response. The coil was placed tangentially to the skull and at an angle of 45° from the midline and held in place by a frame. Participants were lying in a semi-recumbent position and were instructed not to move their heads and to keep their eyes open. MEPs were measured from the abductor digiti minimi muscle by placing two surface Ag/AgCl electrodes in a belly-tendon montage. The active electrode was placed on top of the muscle and the reference electrode on the little finger.

rMT was determined according to established procedures^28,29^. The target area was manually stimulated using single pulses starting at an intensity of 40% of MSO. When there was no MEP, stimulation intensity was increased in steps of 5%. Once the motor hotspot was located, stimulation intensity was decreased in steps of 1% to determine the intensity at which at least 5 out of 10 TMS pulses elicited a MEP with a peak-to-peak amplitude of at least 50 µV. After determination of rMT, spTMS and ppTMS protocols were applied.

spTMS consisted of 50 single pulses at 120% of baseline rMT (defined as the rMT measured in the morning of each treatment period prior to dosing) with a randomized interval between 3.5 and 4.5 s. The spTMS protocol was followed by 50 pairs of pulses in randomized order with inter-stimulus intervals (ISI) of 2, 5, 50, 100, 200, and 300 ms. For ISIs of 2 and 5 ms, conditioning pulses were delivered at 80% of baseline rMT. For all other intervals, conditioning and test pulses were given at an intensity of 120% of baseline rMT.

EMG was measured and recorded with an electroencephalogram (EEG) amplifier (TMSi, Oldenzaal, The Netherlands) with a sample frequency of 2048 Hz. A ground electrode was located between EEG electrode positions Fz and Fpz, as EEG signals were also collected (not analyzed for the current publication). EMG recordings were checked for muscle pre-activation and responses were excluded when muscle activity was greater than 50 µV in the 50 ms prior to the single or conditioning pulse. Customized MATLAB (version R2015a, MathWorks, Natick, MA, USA) routines were used for all analyses. MEPs within 20–45 ms post-spTMS intervals were analyzed post hoc. Peak-to-peak EMG amplitudes were calculated and averaged over 50 repetitions.

For short-interval intracortical inhibition (SICI; 2 and 5 ms ISI), mean peak-to-peak amplitudes of the responses to the 50 unconditioned and 50 conditioned test pulses were calculated. For the unconditioned response, single-pulse responses were evaluated. SICI was calculated as the ratio between conditioned test response (TR) amplitude and the unconditioned response (SP_MEP) amplitude according to the following formula: 100 × TR/SP_MEP (%). For long-interval intracortical inhibition (LICI; 50–300 ms ISI), the mean peak-to-peak amplitude of the responses to the 50 conditioning and 50 test pulses was calculated. LICI was calculated as the ratio between the mean TR amplitude and the mean conditioning response (CR) using the following formula: 100 × TR/CR (%).

### Statistical analysis of the human study

Peak-to-peak MEP amplitude, rMT, SICI, and LICI were analyzed using a mixed model for repeated measures with fixed factors for treatment, period, sequence and treatment by period interaction, and subject nested in sequence as a random effect. The baseline measure for the corresponding outcome was included in the model as a covariate. Estimated treatment effects, two-sided 90% confidence intervals (CI) and *p* values were calculated for measures at 30 min and 2.5 h post-dose. Hochberg’s step-up procedure was used to adjust for multiple testing and Dunnett adjusted *p* values were calculated.

## Results

### TAK-653 increased corticospinal excitability assessed with TMS in rats

We observed a significant increase in corticospinal excitability (as reflected in larger MMG amplitude) with doses of 0.3 mg/kg TAK-653 or higher, compared to vehicle (Fig. 1B, C). In satellite animals, the lowest effective dose resulted in 5.32 ± 0.94 (mean ± standard deviation (SD)) ng/ml TAK-653 in plasma, 1 mg/kg yielded 49.9 ± 35.9 ng/ml, 8 mg/kg yielded 298.2 ± 65.3 ng/ml, and 50 mg/kg resulted in 391.0 ± 190.6 ng/ml (Fig. 1D). Brain concentrations of TAK-653 were 3.53 ± 0.42 ng/g for 0.3 mg/kg, 36.5 ± 24.8 ng/g for 1 mg/kg, 210.6 ± 64.8 ng/g for 8 mg/kg, and 264.2 ± 81.9 ng/g for 50 mg/kg (Fig. 1D). In the animals used for TMS-MMG, a repeated-measures ANOVA revealed a significant effect of dose (F_(5,25)_ = 4.399; *p* = 0.005), time (F_(2.475,61.87)_ = 4.076; *p* = 0.015) and a time × dose interaction (F_(30,150)_ = 1.692; *p* = 0.022). We were unable to observe a dose–response effect with the current data set, yet all effective TAK-653 doses resulted in evoked MMG amplitudes 30–70% higher than those with vehicle.

### Human TMS study participants and pharmacokinetics

Twenty-three males and one female were included. All participants completed the first three study periods (Fig. 2A). Four individuals did not participate in the open-label ketamine period. Demographics are summarized in Table 1. TAK-653 plasma levels at 30 min and 2.5 h post-dose were 0.99 ± 0.94 (mean ± SD) ng/ml and 4.19 ± 0.83 ng/ml for 0.5 mg TAK-653, and 2.57 ± 3.29 ng/ml and 45.99 ± 8.84 ng/ml for 6.0 mg TAK-653, respectively.

**Table 1:** Demographics.

Individuals enrolled	*n*	24
Age, years	Mean (SD)	27.9 (9.0)
	Median	24.5
	Range	20–49
Sex, *n*	Female	1 (4.2%)
	Male	23 (95.8%)
Race, *n*	White	22 (91.7%)
	Asian	1 (4.2%)
	Multiple	1 (4.2%)
Weight, kg	Mean (SD)	79.12 (10.81)
	Median	78.38
	Range	63.8–115.0
Height, cm	Mean (SD)	181.98 (9.88)
	Median	181.50
	Range	158.3–201.2
BMI, kg/m^2^	Mean (SD)	23.92 (2.85)
	Median	23.35
	Range	19.5–29.2

*BMI* body mass index, *SD* standard deviation.

### Safety and tolerability

TAK-653 was well tolerated, and all TAK-653-related adverse events (AEs) were of mild intensity. No serious AEs occurred. In the TAK-653 0.5 and 6 mg dose periods, 37.5% and 50.0% of participants experienced a treatment-emergent AE (TEAE), respectively, compared to 29.2% of participants in the placebo period. The most frequently reported TEAEs after TAK-653 administration were somnolence, headache, and nasopharyngitis (Table 2).

**Table 2:** Most frequent TEAEs (≥5% of individuals in placebo or overall TAK-653).

Preferred term	Participants, *n* (%)			
	Placebo (*n* = 24)	TAK-653 0.5 mg (*n* = 24)	TAK-653 6 mg (*n* = 24)	All TAK-653 (*n* = 24)
Any TEAE	7 (29.2)	9 (37.5)	12 (50.0)	15 (62.5)
Somnolence	2 (8.3)	3 (12.5)	3 (12.5)	6 (25.0)
Headache	2 (8.3)	1 (4.2)	4 (16.7)	4 (16.7)
Nasopharyngitis	0	3 (12.5)	1 (4.2)	4 (16.7)
Oropharyngeal pain	1 (4.2)	0	2 (8.3)	2 (8.3)
Diarrhea	0	1 (4.2)	1 (4.2)	2 (8.3)
Seasonal allergy	0	1 (4.2)	1 (4.2)	2 (8.3)
Fatigue	2 (8.3)	0	1 (4.2)	1 (4.2)

*TEAE* treatment-emergent adverse event.

### TAK-653 increased corticospinal excitability assessed with TMS in humans

No significant effects on peak-to-peak MEP amplitude were observed with 0.5 mg TAK-653 compared to placebo at 30 min and 2.5 h post-dose (*p* = 0.6328, Dunnett adjusted *p* value = 0.8475 at 30 min; *p* = 0.4278, Dunnett adjusted *p* value = 0.6418 at 2.5 h; Fig. 2B, C and Tables 3 and 4). For 6 mg TAK-653 compared with placebo, the effect observed 30 min post-dose was not statistically significant (*p* = 0.0586, Dunnett adjusted *p* value = 0.1053); however, 2.5 h post-dose, a statistically significant increase in MEPs compared to placebo was observed (*p* = 0.0269, Dunnett adjusted *p* value = 0.0497; Fig. 2B, C and Tables 3 and 4). No statistically significant effects were found on change from baseline rMT for TAK-653 0.5 or 6 mg compared with placebo at 30 min or 2.5 h post-dose (Fig. 2D).

**Table 3:** Single-pulse peak-to-peak amplitude.

	Placebo		TAK-653 0.5 mg		TAK-653 6 mg	
	0.5 h Post-dose	2.5 h Post-dose	0.5 h Post-dose	2.5 h Post-dose	0.5 h Post-dose	2.5 h Post-dose
*n*	24	24	24	24	24	23^a^
Change from pre-dose baseline mean (SD) (µV)	−32.81 (756.65)	−139.12 (829.60)	110.32 (845.96)	17.27 (466.26)	260.38 (1132.49)	139.93 (813.97)
Estimate of difference in LS means	NA	NA	99.7	115	411	338
90% CI for difference in LS means	NA	NA	−249, 499	−128, 359	55.5, 766	90.5, 585
*p* Value, Dunnett adjusted *p* Value for TAK-653 vs. placebo	NA	NA	0.633, 0.848	0.428, 0.642	0.059, 0.105	0.027^b^, 0.05^b^

*CI* confidence interval, *LS* least-squares, *NA* not applicable, *SD* standard deviation.

^a^One measurement is missing due to a technical error.

^b^Statistically significant compared to placebo.

**Table 4:** Single-pulse peak-to-peak amplitude raw values.

Mean (SD) single-pulse peak-to-peak amplitude (µV)	Placebo	TAK-653 0.5 mg	TAK-653 6 mg
Pre-dose (baseline)	898.93 (693.15)	841.07 (591.14)	1004.13 (574.60)
0.5 h Post-dose	866.02 (798.62)	951.39 (542.72)	1230.41 (1057.11)
2.5 h Post-dose	759.71 (537.31)	858.33 (516.76)	1101.58 (839.55)

ppTMS responses were evaluated in humans. The changes in magnitude from baseline compared with placebo for LICI using 50, 100, and 200 ms ISIs were not statistically significant for TAK-653 0.5 or 6 mg at 30 min or 2.5 h post-dose. Using a 300 ms ISI, the change from baseline in magnitude of LICI was statistically significant only for TAK-653 0.5 mg at 2.5 h post-dose (an increase revealed by the estimate of difference in least-squares means: 17.2% [90% CI: 5.06%, 29.4%], *p* = 0.0220, Dunnett adjusted *p* value = 0.0406; Table 5). The changes from baseline in the magnitude of SICI were not statistically significant.

**Table 5:** Paired-pulse TMS was not affected by TAK-653 (*n* = 24).

		Placebo		TAK-653 0.5 mg		TAK-653 6 mg	
SICI 2 ms		0.5 h Post-dose	2.5 h Post-dose	0.5 h Post-dose	2.5 h Post-dose	0.5 h Post-dose	2.5 h Post-dose
	Change from pre-dose baseline mean (SD) %	18.35 (57.04)	5.05 (47.57)	0.87 (33.33)	1.77 (35.92)	2.56 (57.34)	−14.58 (37.73)
	Estimate of difference in LS means			−19.7	−5.65	−13.6	−16.1
	90% CI for difference in LS means			−40.6, 1.26	−34.8, 7.64	−34.8, 7.64	−32.5, 0.242
	*p* Value, Dunnett adjusted *p* value for TAK-653 vs. placebo			0.121 (0.210)	0.559 (0.782)	0.288 (0.461)	0.105 (0.183)
SICI 5 ms							
	Change from pre-dose baseline mean (SD) %	28.02 (115.12)	14.30 (81.61)	1.57 (33.19)	4.27 (30.90)	1.36 (81.23)	−7.25 (52.92)
	Estimate of difference in LS means			−33.9	−16.7	−25.8	−18.3
	90% CI for difference in LS means			−69.3, 1.36	−39.4, 6.06	−61.4, 9.8	−41.3, 4.56
	*p* Value, Dunnett adjusted *p* value for TAK-653 vs. placebo			0.113 (0.197)	0.224 (0.369)	0.229 (0.377)	0.185 (0.310)
LICI 50 ms							
	Change from pre-dose baseline mean (SD) %	19.12 (68.54)	0.68 (54.57)	10.93 (56.56)	18.32 (81.44)	16.71 (59.26)	19.40 (110.09)
	Estimate of difference in LS means			−4.67	14.0	1.12	15.1
	90% CI for difference in LS means			−33.3, 24.0	−14.8, 42.8	−27.6, 29.8	13.7, 43.9
	*p* Value, Dunnett adjusted *p* value for TAK-653 vs. placebo			0.787 (0.947)	0.417 (0.626)	0.948 (0.997)	0.383 (0.584)
LICI 100 ms							
	Change from pre-dose baseline mean (SD) %	19.37 (98.14)	0.15 (29.16)	12.31 (28.81)	5.91 (18.89)	−8.53 (58.03)	−28.64 (142.56)
	Estimate of difference in LS means			−9.50	−0.326	−14.9	3.71
	90% CI for difference in LS means			−38.6, 19.6	−7.23, 6.58	−44.3, 14.6	−3.32, 10.7
	*p* Value, Dunnett adjusted *p* value for TAK-653 vs. placebo			0.582 (0.802)	0.937 (0.995)	0.398 (0.604)	0.379 (0.582)
LICI 200 ms							
	Change from pre-dose baseline mean (SD) %	7.44 (30.26)	2.88 (21.25)	15.01 (47.77)	9.90 (42.58)	4.64 (32.12)	11.54 (30.16)
	Estimate of difference in LS means			7.67	7.36	−2.59	9.36
	90% CI for difference in LS means			−3.96, 19.3	−3.44, 18.2	−14.2, 9.04	−1.45, 20.2
	*p* Value, Dunnett adjusted *p* value for TAK-653 vs. placebo			0.274 (0.439)	0.258 (0.417)	0.709 (0.909)	0.152 (0.259)
LICI 300 ms							
	Change from pre-dose baseline mean (SD) %	3.25 (46.44)	−10.0 (27.72)	6.51 (44.03)	8.62 (36.05)	0.05 (35.65)	−18.03 (33.48)
	Estimate of difference in LS means			1.15	17.2	−6.28	9.58
	90% CI for difference in LS means			−16.3, 18.6	5.06, 29.4	−23.7, 11.2	−2.59, 21.8
	*p* Value, Dunnett adjusted *p* value for TAK-653 vs. placebo			0.912 (0.991)	0.022 (0.041)	0.547 (0.769)	0.193 (0.321)

## Discussion

TMS-evoked motor responses were enhanced by TAK-653 in both rats and humans at similar plasma concentrations. In rats, we observed no effect with a dose of 0.1 mg/kg and an increase in MMG amplitude with doses of 0.3 mg/kg or higher, corresponding to 5.32 ng/ml or higher in plasma. TAK-653 was detected in the brains after the procedures, indicating the compound crossed the blood–brain barrier. In healthy humans, a single dose of TAK-653 6 mg, corresponding to a mean plasma level of 45.99 ng/ml at expected *t*_max_, significantly increased MEP amplitude from baseline compared to placebo. TAK-653 0.5 mg, corresponding to a mean plasma level of 4.19 ng/ml at expected *t*_max_, did not elicit an effect on MEP amplitude. There was no change in rMT with either dose of TAK-653. In addition, the only ppTMS assay that revealed a difference in TAK-653 from placebo in humans was with LICI at ISI 300 ms, but the difference was with the low dose that did not induce changes in spTMS.

These results indicate that noninvasive brain stimulation can be used to generate translational neurocircuitry biomarkers that capture subtle modulation of glutamate synaptic activity. TMS of the primary motor cortex is likely activating a cortical column and its projection to the spinal cord motoneurons that drive the response in the activated muscle. As neuromuscular junction synapses utilize acetylcholine as their neurotransmitter and TAK-653 has demonstrated in vitro selectivity for AMPA receptors, the change in MEP by TAK-653 should be driven by CNS effects. Further in support of a glutamate receptor-mediated effect, only evoked responses elicited by spTMS rather than the ppTMS metrics that capture modulation of cortical inhibition^21^ were altered by TAK-653. Thus, our data reveal that TAK-653 modulates corticospinal excitability in a healthy brain and indicate that neurostimulation approaches, such as TMS, can be applied as biomarkers to capture modulation of glutamate synaptic activity. Our ppTMS studies should be interpreted with caution, however. In order to minimize the duration of the procedure in the human study and driven by technical limitations in the rat study, we chose to test SICI and LICI, excluding intracortical facilitation (ICF). SICI and LICI are thought to capture intracortical inhibitory processes and ICF is related to glutamate activity^21^, therefore we chose to focus ppTMS on inhibition-related measures.

The effect of TAK-653 on corticospinal excitability assessed with MEPs supports the use of neurostimulation as a biomarker but does not necessarily mean that this compound will restore circuitry function in depression. The motor cortex is neither anatomically nor functionally involved in the regulation of emotional behavior in humans. However, a large body of data implicates the frontal-striatal circuitry that includes the dorsolateral prefrontal cortex (DL-PFC), subgenual anterior cingulate cortex, amygdala, and ventral striatum in mood disorders^30^. Furthermore, impaired functional connectivity of the DL-PFC with emotion-related circuits has been identified in MDD patients^31^, and EEG signals evoked by DL-PFC TMS differ in MDD patients from controls^20^. In line with these observations, devices for repeated TMS of the DL-PFC have been approved and a range of TMS protocols are rapidly expanding as therapeutic options for treatment-resistant depression^32,33^. Thus, DL-PFC TMS, perhaps coupled with EEG to record TMS-evoked regional potentials, can be used to assess the effect of ketamine, TAK-653 or any novel glutamate-based antidepressant on disease-related circuitry dysfunction in MDD patients.

The study was performed with great caution as both TMS and AMPA receptor potentiation might theoretically increase the risk of seizures by potentiating glutamatergic synapses^26^. We did not observe any evidence of seizures or convulsions and there were no dose-related AEs and no serious AEs. TAK-653 was generally well tolerated by the participants, supporting further development of TAK-653.

Some caveats with our study are worth addressing. We tried to reproduce stimulation parameters and data collection as much as possible in rats and humans. However, in order to minimize stress and movement artifacts while allowing reliable muscle response in rats, we aimed for light anesthesia combined with a noninvasive method to record muscle responses. Thus, TMS-MMG was employed given it is a reliable surrogate for TMS-EMG^25^. Baseline TMS-MMG could not be obtained in rats because of the need of oral dosing 2 h prior to the measures of interest. Therefore, the comparisons were made between TAK-653 and vehicle, and any difference in baseline responses could not be identified. TAK-653 increased MMG amplitude, and any effect of the anesthetic was controlled by inclusion of vehicle-treated animals. Another potential concern is the high variability of human TMS–EMG data; however, this variability was within the expected range. The fact that a significant difference from placebo was observed with our higher dose despite such variability reinforces the conclusion that TAK-653 increased circuitry excitability. In addition, we used baseline rMT to guide stimulation intensity for all measurements. Because adjusting the TMS intensity to compensate for post-drug changes in rMT can change the outcome^34^, and because the rat TMS study used the same stimulation intensity in all treatment groups, we chose not to adjust the TMS strength. We did not observe changes in rMT with treatment, so it is unlikely that any adjustment would have revealed a different outcome. Lastly, we omitted testing ICF to minimize patient burden. This decision resulted in not having a pTMS paradigm related to intracortical glutamate function^21^ and MEP data being the only direct assessment of excitatory neurotransmission.

In conclusion, our data represent an important step forward because they provide evidence of a noninvasive, translational modulation of physiological outcomes of a glutamate-based neural circuit in a healthy brain. Methodologies such as quantitative EEG or magnetic resonance spectroscopy could be considered to measure subtle AMPA receptor modulation in humans, but they miss the detection of functional outcomes of brain circuit activation. Our data show that TMS-evoked motor responses can detect discrete changes in cortical excitability in a defined neural circuit, enabling pharmacological assessments of glutamatergic CNS activity in early drug development. To our knowledge, this is the first demonstration of a circuitry biomarker sensitive to direct positive modulation of AMPA receptors being modulated in a similar manner in rodents and humans.
